# Supplementary material for: Impacts of past abrupt land change on local biodiversity globally
Source: Nat Commun. 2019 Dec 2;10:5474. doi: 10.1038/s41467-019-13452-3 (PMC6888856; doi:10.1038/s41467-019-13452-3)
Supplement: Supplementary file 1 — Supplementary Information [file 41467_2019_13452_MOESM1_ESM.pdf]

Supplementary Information

Impacts of past abrupt land change on local biodiversity globally

Jung et al.

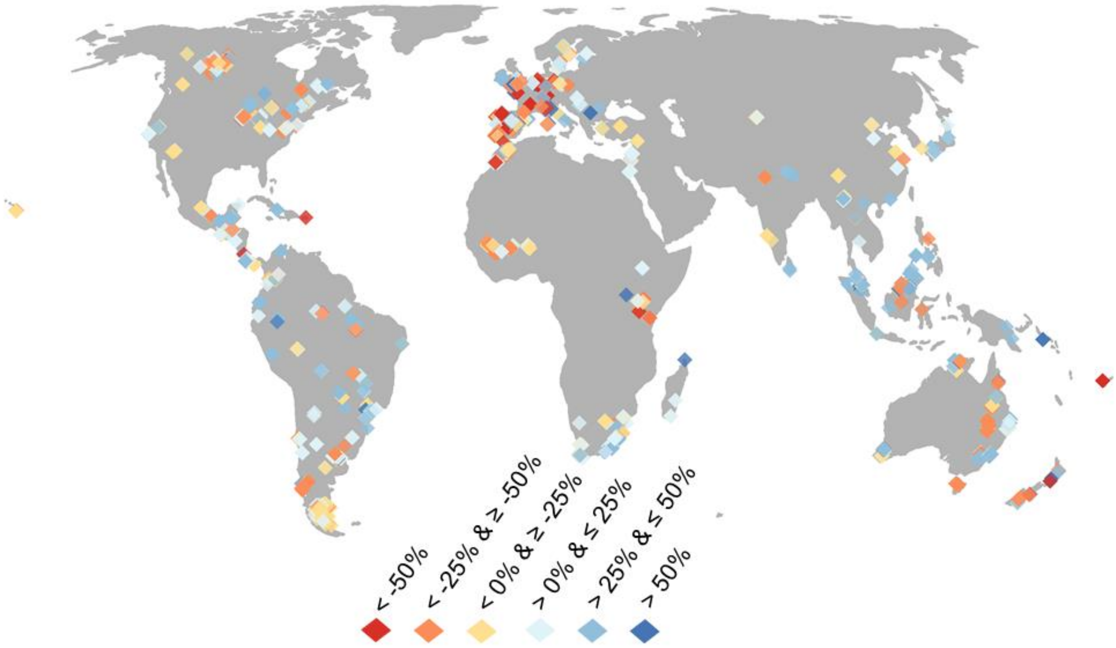

Supplementary Figure 1: Location of sites with an abrupt land change with shift in trend. Location of 5,563 sites from 377 studies in the PREDICTS database[36] with an abrupt land change in the monitoring period (since 1982) of the Landsat 4-8 missions with a shift in trend. Colours indicate negative or positive differences in EVI trend (Supplementary Figure 2b). For ease of viewing, the locations of 10,102 sites without an abrupt land change have been omitted. Map shown in Eckert IV equal-area projection.

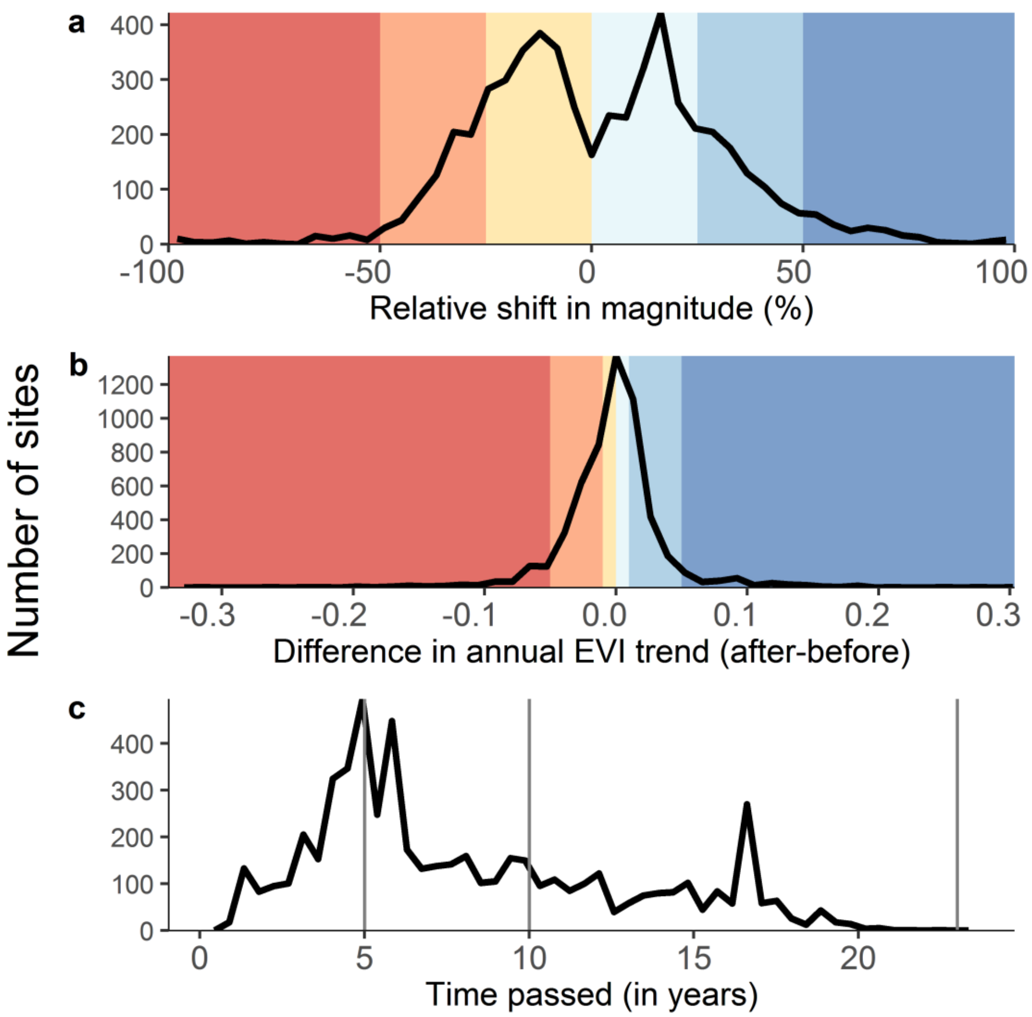

Supplementary Figure 2: Number of sites with abrupt land change per attribute. Number of sites (black line) per attribute of abrupt land change with (**a**) the relative shift in magnitude, (**b**) the shift in trend as difference in annual EVI trend, and (**c**) the time passed between abrupt land change and biodiversity sampling. Background colours in (**a**) and (**b**) indicate the binning into six groups for shifts in magnitude (> 50 %, > 25 % to ≤ 50 %, and ≤ 25 % EVI loss [--- to -] or gain [+++ to +]), and in trend (0.01, 0.05, and > 0.05 annual negative [--- to -] to positive [+++ to +] EVI trend differences). Gray lines in (**c**) delineate bins of time passed (≤ 5 years, > 5 and ≤ 10 years, and >10 years). Colours as in Figure 2.

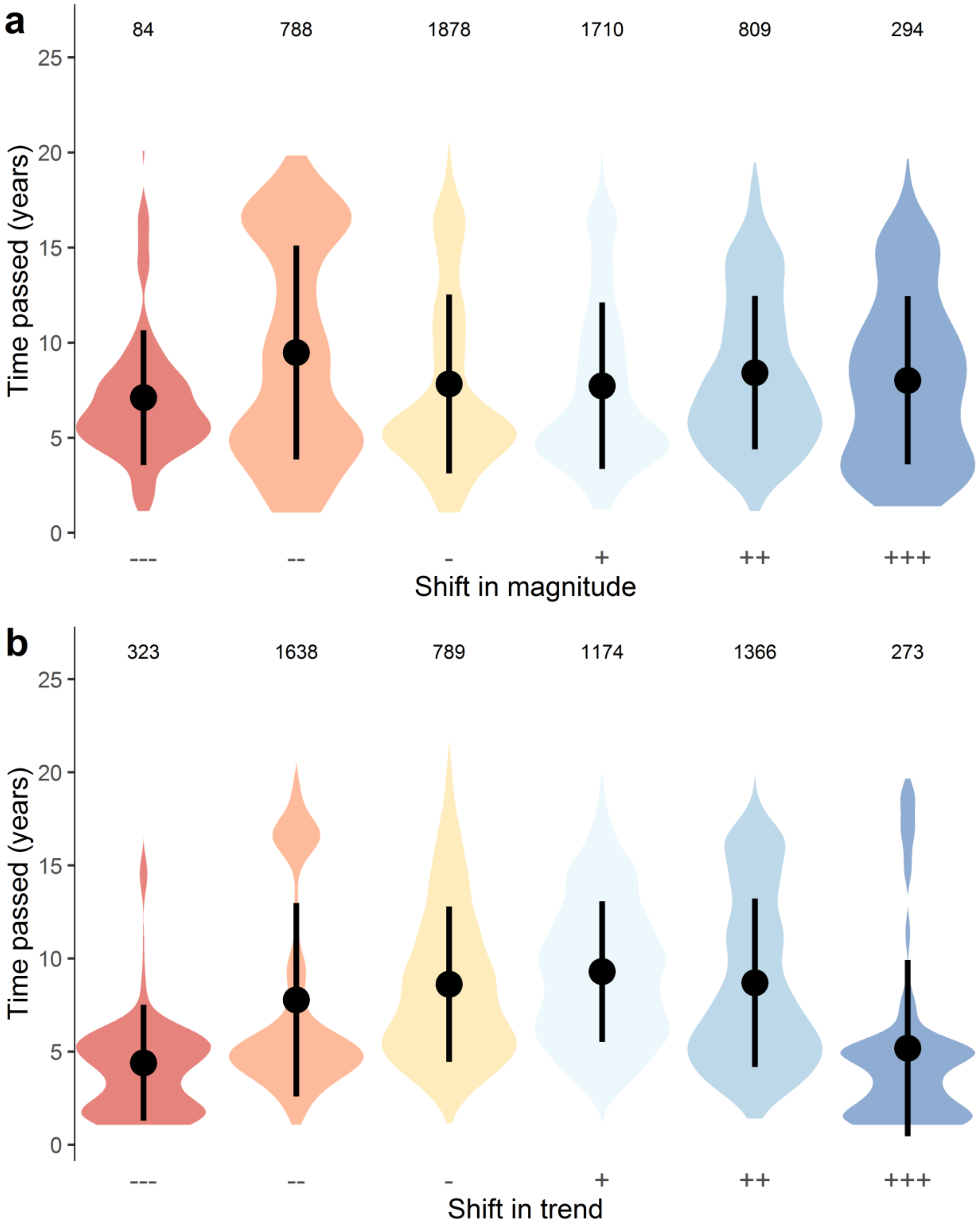

Supplementary Figure 3: Distribution of time passed between abrupt land changes and start of biodiversity sampling. Shown for (**a**) shifts in magnitude, and (**b**) shift in trend bins. Colours as in Figure 2. Black dots and error bars show the mean ± one standard deviation. Number of sampled sites per bin are shown above each bin. Source data are provided as a Source Data file.

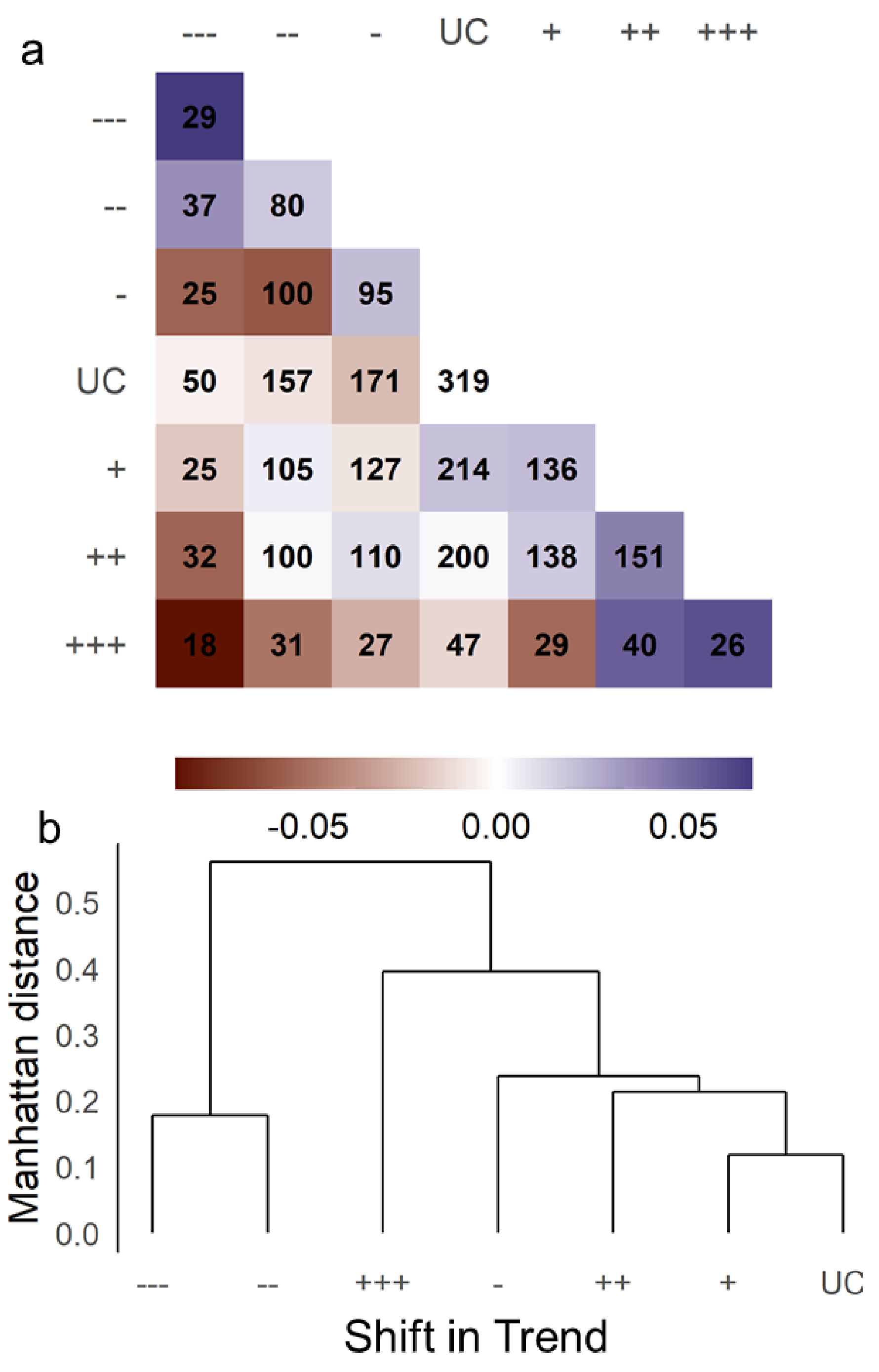

Supplementary Figure 4: Mean similarity in species assemblage composition (Sørensen similarity index) between pairs of sites with a shift in trend. Calculated as average similarity within the same study and land-use category without (UC) and with an abrupt land change with varying shifts in EVI trend (**a**). Colours indicate whether similarity of species assemblages was on average greater (purple) or smaller (brown) relative to unchanged sites. Symbols indicate positive (+++, ++, +) or negative (---, --, -) shifts in trend of annual EVI (see Methods).

Numbers in (**a**) indicate the total number of studies for which pairwise comparisons between sites could be made. (**b**) Dendrograms show hierarchical clustering of all pairwise similarities based on the average Manhattan distance between pairs of sites; sites with more similar assemblage composition are in branches of closer proximity.

a

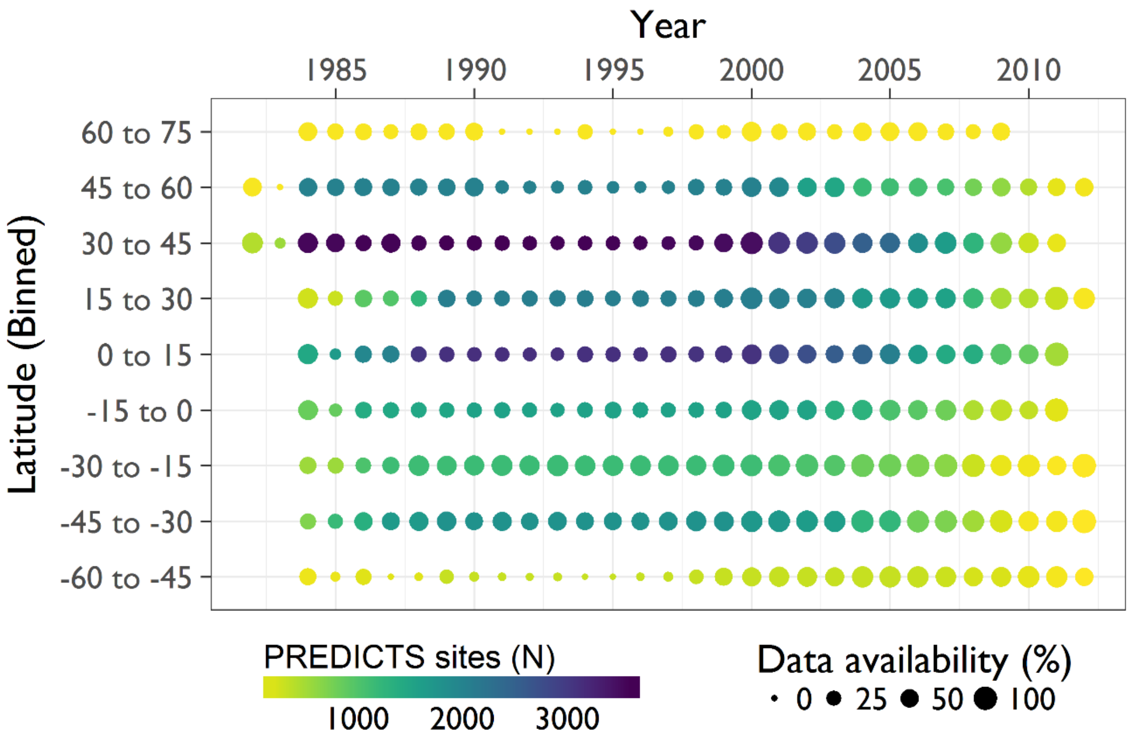

b

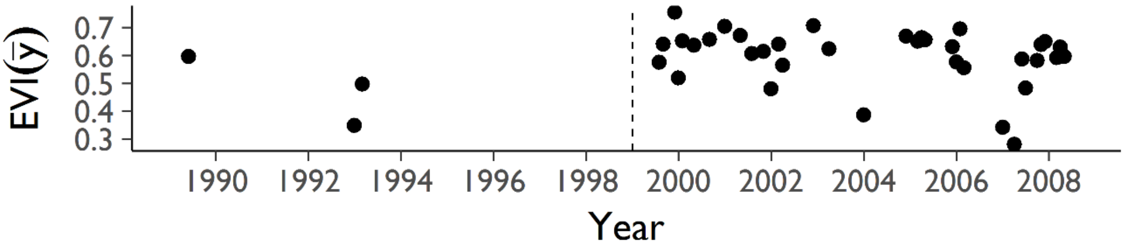

Supplementary Figure 5: Average temporal distribution of Landsat data and an example times series of Landsat data. (**a**) Distribution of available Enhanced Vegetation Index (EVI) data in years covered by the Landsat missions. Points show the average monthly EVI data availability per year (0 to 12 months of data) across time series and PREDICTS sites grouped by 15˚ latitude bins. The size of points indicates the mean data availability (0 to 100 % with 100 % having 12 months of available data in a given year), while the colour shows the number of PREDICTS sites contributing to the mean (as PREDICTS sites were sampled in varying years). (**b**) Example time series for one PREDICTS site with a high proportion of missing data before 1999. In all analyses such time series were truncated to the period from 1999 onwards (indicated by the dashed line).

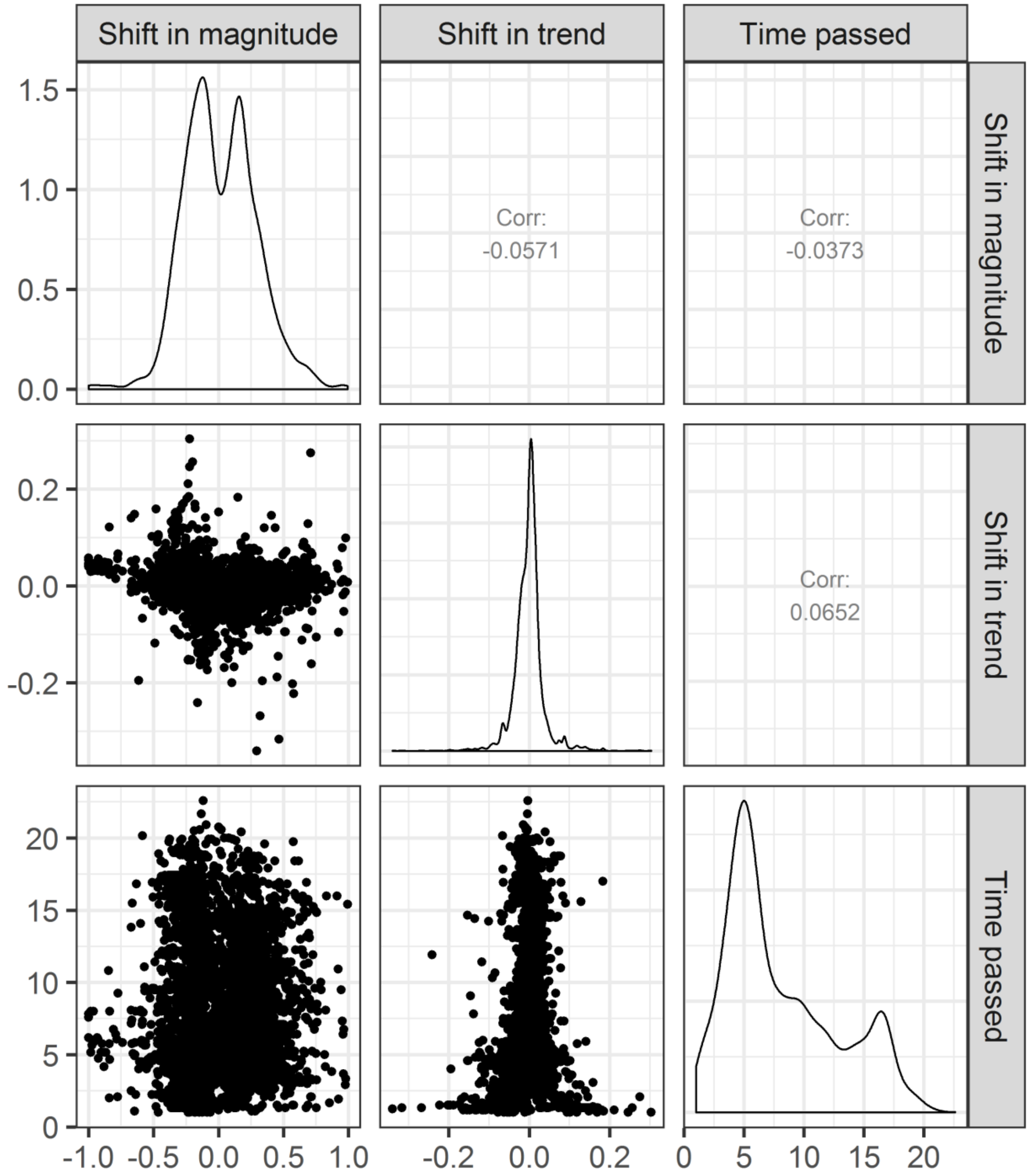

Supplementary Figure 6: Correlations between attributes of abrupt land change. Showing shifts in magnitude, trend and time passed (see Methods). The lower facets show a point density plot, the upper facets the Pearson correlation coefficient between pairs of attributes and the diagonal a density plot.

Supplementary Table 1: Number of PREDICTS sites and studies with an abrupt land change. Shown as either a change in magnitude (columns) and/or change in trend (trend). Symbols as in Figure 2.

Shift in magnitude

Shift in trend		- - -	- -	-	UC	+	+ +	+ + +	Total sites	Studies
	**- - -**	2	8	192	NA	73	26	22	323	57
	**- -**	7	281	642	NA	497	158	53	1638	175
	**-**	7	88	256	NA	231	154	53	789	184
	**UC**	NA	NA	NA	10102	NA	NA	NA	10102	358
	**+**	9	102	399	NA	410	205	49	1174	237
	**+ +**	47	172	342	NA	465	254	86	1366	224
	**+ + +**	12	137	47	NA	34	12	31	273	56
	**Total sites**	84	788	1878	10102	1710	809	294		
	**Studies**	34	135	246	358	263	171	83		
