# Supplementary material for: Impacts of past abrupt land change on local biodiversity globally
Source: Nat Commun. 2019 Dec 2;10:5474. doi: 10.1038/s41467-019-13452-3 (PMC6888856; doi:10.1038/s41467-019-13452-3)
Supplement: Supplementary file 3 — Reporting Summary [file 41467_2019_13452_MOESM3_ESM.pdf]

nature research

Corresponding author(s): Martin Jung

Last updated by author(s): Oct 27, 2019

Reporting Summary

Nature Research wishes to improve the reproducibility of the work that we publish. This form provides structure for consistency and transparency in reporting. For further information on Nature Research policies, see Authors & Referees and the Editorial Policy Checklist .

Statistics

For all statistical analyses, confirm that the following items are present in the figure legend, table legend, main text, or Methods section.

n/a	Confirmed	
☐	☒	The exact sample size (*n*) for each experimental group/condition, given as a discrete number and unit of measurement
☐	☒	A statement on whether measurements were taken from distinct samples or whether the same sample was measured repeatedly
☐	☒	The statistical test(s) used AND whether they are one- or two-sided<br>*Only common tests should be described solely by name; describe more complex techniques in the Methods section.*
☐	☒	A description of all covariates tested
☐	☒	A description of any assumptions or corrections, such as tests of normality and adjustment for multiple comparisons
☐	☒	A full description of the statistical parameters including central tendency (e.g. means) or other basic estimates (e.g. regression coefficient) AND variation (e.g. standard deviation) or associated estimates of uncertainty (e.g. confidence intervals)
☐	☒	For null hypothesis testing, the test statistic (e.g. *F*, *t*, *r*) with confidence intervals, effect sizes, degrees of freedom and *P* value noted<br>*Give P values as exact values whenever suitable.*
☒	☐	For Bayesian analysis, information on the choice of priors and Markov chain Monte Carlo settings
☐	☒	For hierarchical and complex designs, identification of the appropriate level for tests and full reporting of outcomes
☐	☒	Estimates of effect sizes (e.g. Cohen's *d*, Pearson's *r*), indicating how they were calculated

Our web collection on statistics for biologists contains articles on many of the points above.

Software and code

Policy information about availability of computer code

Data collection	All analysis was done using publicly available R packages with version numbers stated in the methods and on the publicly available repository. Remote sensing data collection and preparation was done using Google Earth Engine.
Data analysis	Data and code to reproduce the results and main figures is made available in a GitHub repository (https://github.com/Martin-Jung/PastDisturbance).

For manuscripts utilizing custom algorithms or software that are central to the research but not yet described in published literature, software must be made available to editors/reviewers. We strongly encourage code deposition in a community repository (e.g. GitHub). See the Nature Research guidelines for submitting code & software for further information.

Data

Policy information about availability of data

All manuscripts must include a data availability statement. This statement should provide the following information, where applicable:

- Accession codes, unique identifiers, or web links for publicly available datasets
- A list of figures that have associated raw data
- A description of any restrictions on data availability

The PREDICTS biodiversity data are publicly available in the Natural History Museum Data Portal (DOI: doi.org/10.5519/0066354). All remote sensing data are accessible via Google Earth Engine (earthengine.google.com) and pre-processed time series are deposited on GitHub (https://github.com/Martin-Jung/PastDisturbance).

Field-specific reporting

Please select the one below that is the best fit for your research. If you are not sure, read the appropriate sections before making your selection.

☐ Life sciences ☐ Behavioural & social sciences ☒ Ecological, evolutionary & environmental sciences

For a reference copy of the document with all sections, see nature.com/documents/nr-reporting-summary-flat.pdf

Ecological, evolutionary & environmental sciences study design

All studies must disclose on these points even when the disclosure is negative.

Study description	This study investigates the pervasive impacts of past abrupt land changes on local biodiversity globally and the potential for biodiversity to recover after >10 years. We used a novel framework to quantitatively and reproducibly evaluate abrupt land change, using remotely-sensed Landsat-derived time series of photosynthetic activity of high spatial resolution (~30m) and long temporal duration (1982 to 2015), and its impacts on biodiversity, as observed by the PREDICTS dataset.
Research sample	This study uses data from the PREDICTS project, a taxonomically- and geographically-broad dataset representing over 2% of all described terrestrial taxa (3.2 million records sampled at over 26,000 locations representing over 47,000 species).
Sampling strategy	Not applicable. Differences in sampling groups and methodology are accounted for using a hierarchical modeling approach.
Data collection	Data was previously collected and published as part of the PREDICTS project (Hudson et al. 2017) in which authors of ecological published studies were contacted and asked for the raw data underlying their study. Differences among study methodology, sampling design and local land use were accounted for by using a hierarchical model that tests for the difference in local biodiversity between sites with and sites without a past abrupt land change.
Timing and spatial scale	Various spatial and temporal scales as indicated in the manuscript and previous analyses (Newbold et al. 2015, Hudson et al. 2017). Only sites with given satellite records prior to biodiversity sampling were considered with the spatial scale incorporated in hierarchical modeling and the extraction of remote sensing data.
Data exclusions	Exclusion of specific sites unsuitable for the analysis as indicated in the methods. This particularly includes a single study with large biomass outliers and additional care was taken for studies where positional accuracy was assumed to be low.
Reproducibility	All data underlying this study is publicly available. Preprocessed data and code to reproduce the analysis are made available.
Randomization	No randomization was done or part of the data collection. PREDICTS distinguished between studies, spatial blocks, sites and local land use which we accounted for in our hierarchical modeling approach.
Blinding	Not applicable. Data was acquired as part of the data curation process of the PREDICTS project.

Did the study involve field work? ☐ Yes ☒ No

Reporting for specific materials, systems and methods

We require information from authors about some types of materials, experimental systems and methods used in many studies. Here, indicate whether each material, system or method listed is relevant to your study. If you are not sure if a list item applies to your research, read the appropriate section before selecting a response.

Materials & experimental systems

n/a	Involved in the study	
☒	☐	Antibodies
☒	☐	Eukaryotic cell lines
☒	☐	Palaeontology
☒	☐	Animals and other organisms
☒	☐	Human research participants
☒	☐	Clinical data

Methods

n/a	Involved in the study	
☒	☐	ChIP-seq
☒	☐	Flow cytometry
☒	☐	MRI-based neuroimaging
